# Transarterial Chemoembolization Combined With Radiofrequency Ablation Versus Hepatectomy for Hepatocellular Carcinoma: A Meta-Analysis

**DOI:** 10.3389/fsurg.2022.948355

**Published:** 2022-07-11

**Authors:** Yuan Dan, Wenjun Meng, Wenke Li, Zhiliang Chen, Yongshuang Lyu, Tianwu Yu

**Affiliations:** ^1^ Department of Hepatobiliary Surgery, Yongchuan Hospital, Chongqing Medical University, Chongqing, China; ^2^ Department of Gastrointestinal Surgery, Yongchuan Hospital, Chongqing Medical University, Chongqing, China; ^3^ Department of Biotherapy, Cancer Center, West China Hospital, Sichuan University, Chengdu, China

**Keywords:** radiofrequency ablation, hepatectomy, transarterial chemoembolization, overall survival, disease-Free survival, major complications

## Abstract

**Background:**

Although many studies reported the effectiveness of transarterial chemoembolization (TACE) combined with radiofrequency ablation (RFA) or surgical resection (SR) in the treatment of hepatocellular carcinoma (HCC), the efficacy of these two strategies remains controversial. Therefore, this meta-analysis aimed to evaluate and compare the efficacy of sequential use of TACE plus RFA (TACE + RFA) and SR alone in treating HCC.

**Methods:**

Relevant studies with unmatched and propensity score-matched patients were identified by comprehensive search of MEDLINE, PubMed, EMBASE, Web of Science, and Cochrane electronic databases. Meta-analysis was conducted using Review Manager (RevMan) software version 5.4.1. Finally, 12 eligible studies were included in this study, including 11 case–control studies and 1 randomized controlled trial. The primary outcome of interest for this study was to compare the 1-, 3-, and 5-year overall survival (OS) and disease-free survival (DFS), major complications, 5-year OS in different tumor diameters between the two treatment strategies, and hospital stay time.

**Results:**

HCC patients who received TACE + RFA had a lower incidence of complication rates and shorter hospital stay time than those who received SR alone. Among these studies using propensity score-matched cohorts, SR had better 3- and 5-year OS than TACE + RFA, whereas there were no significant differences between TACE + RFA and SR regarding the 1-, 3-, and 5-year DFS. When the tumor diameter is longer than 3 cm, the 5-year OS rate is better when SR is selected.

**Conclusion:**

There was no significant difference in the short-term survival outcomes between TACE + RFA and SR in HCC patients. Moreover, SR is superior to TACE + RFA in terms of long-term beneficial effects but may result in a higher risk of major complications and a longer hospital stay time.

## Introduction

Hepatocellular carcinoma (HCC) is the second leading cause of cancer mortality worldwide, with the fifth-highest incidence in men and ninth-highest incidence in women (1). There is a high incidence of HCC in China, which may be due to China's birth control policy, limited oral contraceptive use, and especially the high incidence of hepatitis B virus (2). Surgical resection (SR) has always been regarded as the best treatment for HCC, but the long-term survival remains unsatisfactory due to the high recurrence rate (3). In addition to SR, transarterial chemoembolization (TACE) has been widely used in recent years as a standard treatment for patients with intermediate-stage HCC. The advantage of TACE is to selectively or nonselectively insert the catheter into the targeted artery that supplies blood to the tumor. The target artery is then occluded by injecting appropriate embolic drugs, resulting in ischemic necrosis of tumor tissues. On the other hand, radiofrequency ablation (RFA) is also as effective as surgery for very early HCC (single nodule with a diameter ≤2.0 cm), especially in local disease control and sustained survival (4). A study has shown that TACE with RFA (abbreviated as TACE + RFA in this article) is not only safe and effective in the treatment of HCC but also can delay tumor progression and improve long-term effects (5).

However, there is controversy regarding the effectiveness between TACE + RFA and SR in treating HCC. Some studies demonstrated that HCC patients receiving SR have a better prognosis than TACE + RFA (6), while others concluded that TACE + RFA yielded a better prognosis (7, 8). Therefore, the aim of this meta-analysis was to evaluate and compare the short- and long-term survival outcomes and major complications between TACE + RFA and SR alone in the treatment of HCC.

## Methods

### Search Strategy

This meta-analysis was conducted according to the PRISMA (Preferred Reporting Items for Systematic Reviews and Meta-Analyses) guideline (9). Relevant studies from January 1, 2008 to December 31, 2021 were systematically searched and retrieved from MEDLINE, PubMed, EMBASE, Web of Science, and Cochrane electronic databases. Search terms (including related variants and abbreviations) and their combinations used in this search strategy included “radiofrequency ablation,” “TACE,” “transcatheter arterial chemoembolization,” “liver cell carcinoma,” “hepatoma”, “liver resection,” and “hepatectomy.” The searching process was performed independently by three authors. Studies cited as references were also considered potentially relevant articles for this study and were further retrieved and evaluated by the three authors.

### Selection Criteria and Data Extraction

Studies included in this meta-analysis met the following inclusion criteria: (I) the studies were cohort studies, observational studies, or randomized controlled trials (RCTs); and (II) the patients received TACE + RFA or SR intervention. There were no restrictions on race, sex, or age of the patients included in the literature searched. The retrieved studies were excluded when they met the following criteria: (I) meta-analyses, conference abstracts, review articles, or case reports/series; (II) studies with insufficient information on the survival outcomes [overall survival (OS) or disease-free survival (DFS)] and complications; and (III) studies with similar results using the same cohort from the same center. All eligible studies were independently reviewed and cross-checked by two authors. In case of disagreement, the dispute was resolved by discussion to reach a consensus or judged by an independent researcher. The content of the data extraction included the name of the first author, year of publication, sample size, demographics and clinical data of the subjects, as well as the treatment outcomes.

### Quality Assessment of the Included Studies

The quality of the included studies was evaluated using the Newcastle–Ottawa scale (NOS). After the NOS assessment, a study with a NOS score of less than four points is considered a low-quality study, while a study with a NOS score of more than seven points is considered high-quality research.

### Statistical Analysis

This meta-analysis was conducted using Review Manager (RevMan) software version 5.4.1. The degree of heterogeneity of the included studies was assessed. If there was heterogeneity (*I*^2^* *>* *50% or *P *< 0.1), a random-effects model (D–L method) was used; otherwise, a fixed-effects model (M–H method) was used. The odds ratios (OR) and 95% confidence interval (CI) of the included study were obtained and presented. A *P*-value <0.05 is considered statistically significant. Publication bias of the literature was evaluated using the Egger test of Stata 14.0 software. If *P *> 0.05, there was no publication bias. In addition, OS, DFS, incidence of major complications, and hospital stay time in these literature works were recorded and collated.

## Results

### Literature Search Results and Included Research Status

**Figure 1:**
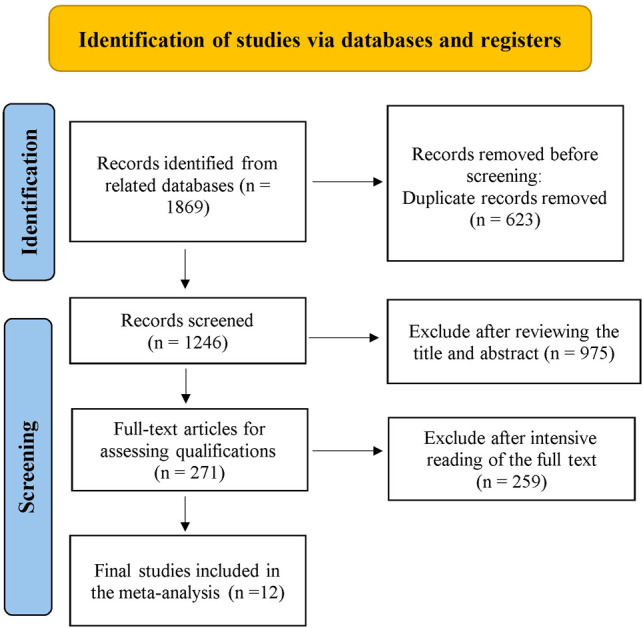
Article selection process flow chart.

Relevant studies were screened based on our search strategy, and the data from these studies were retrieved. Figure 1 shows the flow diagram of the search process for retrieving eligible studies. Finally, 12 studies that met the inclusion and exclusion criteria were included and assessed in this meta-analysis, including 11 case–control studies and 1 RCT. Table 1 shows the characteristics and information of the 12 included studies.

**Table 1:** Characteristics of included studies.

Author	Year	Type of study	Number		Median age	Sex (male/ female)	Median tumor diameters (cm)	Child–Pugh class	NOS score
			TACE + RFA	SR				(A/B/C)	
Kuo et al. (10)	2021	CS	176	125	63.3	196/105	179/122 (<3.0/>3.0)	284/17/0	8
Lin et al. (6)	2020	CS	57	140	62.6	160/37	7.42	178/19/0	6
Lee et al. (11)	2019	CS	60	139	60.3	158/41	3.74	196/3/0	7
Peng et al. (12)	2018	CS	107	79	56.2	91/11	2.57	173/13/0	7
Pan et al. (13)	2017	CS	206	214	54.7	393/27	83/252/85 (≤3/3–5/>5 cm)	402/18/0	7
Lee et al. (14)	2017	CS	70	84	60.8	116/38	2.56	150/4/0	6
Bholee et al. (15)	2017	CS	74	148	53.1	204/18	2.90	214/8/0	8
Liu et al. (8)	2016	RCT	100	100	50.5	180/20	2.90	194/6/0	8
Takuma et al. (16)	2013	CS	154	176	68.9	235/95	2.30	283/47/0	7
Kim et al. (17)	2013	CS	37	47	60.1	67/17	3.60	82/2/0	5
Kagawa et al. (18)	2010	CS	62	55	66.8	79/38	2.60	117/0/0	5
Yamakado et al. (19)	2008	CS	104	62	65.8	130/36	2.60	166/0/0	6

*CS, cohort study; RCT*, *randomized controlled trial; RFA, radiofrequency ablation; SR, surgical resection; TACE, transarterial chemoembolization; NOS, Newcastle–Ottawa scale (NOS).*

### Meta-Analysis

#### One-Year OS

**Figure 2:**
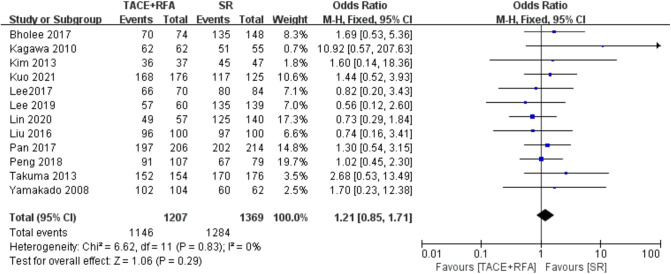
Comparison of 1-year overall survival (OS).

Twelve studies reported 1-year OS. Since there was no heterogeneity among the literature selected (*I*^2^ = 0%, *P *= 0.830), a fixed-effects model was used for meta-analysis. As shown in Figure 2, there was no significant difference between the TACE + RFA group and the SR group regarding 1-year OS (OR = 1.210, 95% CI = 0.850–1.710, Z = 1.060, *P *= 0.290).

#### Three-Year OS

All 12 eligible studies reported 3-year OS, but the heterogeneity test revealed significant differences among these studies (*I*^2^* *= 54%, *P *= 0.010). It is suspected that the heterogeneity within the 12 studies may be due to covariables such as patient characteristics, risk factors, and tumor characteristics. Therefore, the random-effects meta-analysis was conducted, and the results showed that there is no significant difference between HCC patients who received the TACE + RFA group and those who received the SR group regarding the 3-year OS (OR = 0.850, 95% CI = 0.630–1.160, Z = 1.010, *P *= 0.310).

**Figure 3:**
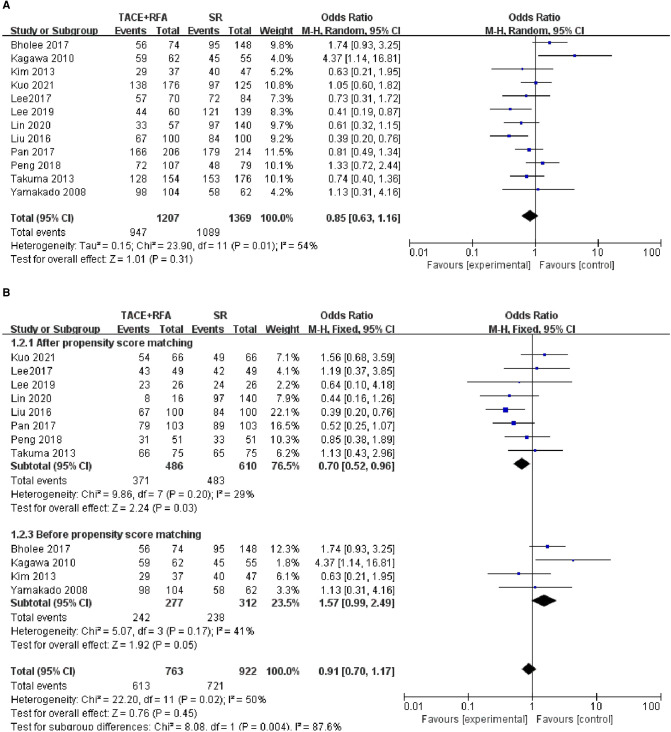
Comparison of (**A**) 3-year OS, (**B**) 3-year overall survival (OS) using propensity score-matched.

Of the 12 studies, 8 studies were conducted using the propensity score-matched cohorts. Moreover, there was no heterogeneity among these studies (*I*^2^* *= 29%, *P *= 0.200). Therefore, fixed-effects meta-analysis was performed. However, the results showed that HCC patients who received TACE + RFA had significantly better 3-year OS than those who received SR alone (OR = 0.700, 95% CI = 0.520–0.960, Z = 2.240, *P *= 0.030). The results of the two meta-analyses are shown in Figure 3.

#### Five-Year OS

Of the 12 eligible studies, 11 studies reported 5-year OS and were included in the following analysis. There was significant heterogeneity among the 11 included studies (*I*^2^*^ ^*= 46%, *P *= 0.050), indicating that different covariables in these studies may contribute to the heterogeneity. Therefore, a random-effects propensity score model was performed. The results showed that HCC patients who received SR had significantly better 5-year OS compared with those who received TACE + RFA (OR = 0.620, 95% CI = 0.490–0.790, Z = 3.910, *P *< 0.0001).

**Figure 4:**
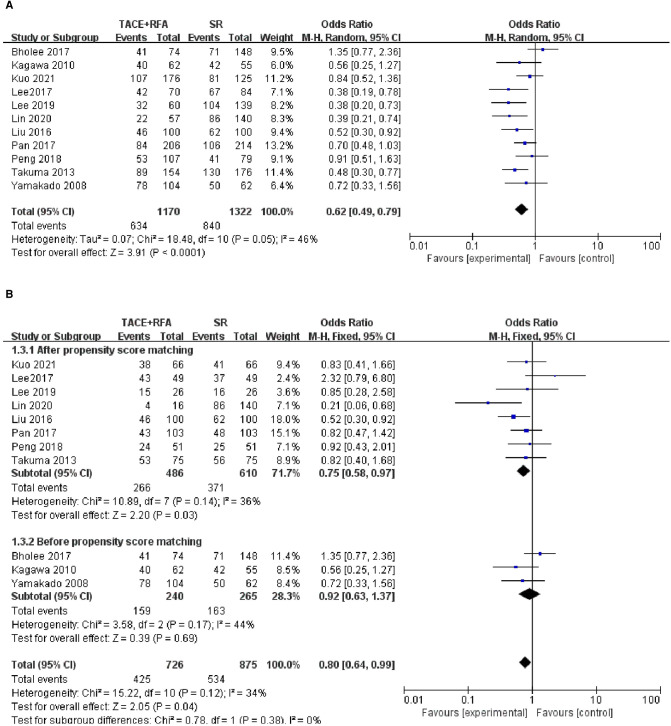
Comparison of (**A**) 5-year OS, (**B**) 5-year OS using propensity score-matched.

Of the 11 studies, 8 studies were conducted using the propensity score-matched cohorts, and there was no heterogeneity among these studies (*I*^2^* *= 36%, *P *= 0.140). Therefore, a fixed-effects model was chosen to combine the effect size. Meta-analysis results showed that 5-year OS in the SR group was higher than that in the TACE + RFA group (OR = 0.750, 95% CI = 0.580–0.970, *Z* = 2.200, *P *= 0.030). The above results are shown in Figure 4.

#### One-Year DFS

Among the 12 studies, only 6 studies reported 1-year DFS and were then included in the following meta-analysis. The results of the heterogeneity test indicated that there was heterogeneity among the included studies (*I*^2^* *= 53%, *P *= 0.060), suggesting that different covariables among the studies contributed to heterogeneity. Therefore, random-effects meta-analysis was conducted, and it showed that there was no significant difference in 1-year DFS between patients who received TACE + RFA and those who received SR alone (OR = 1.150, 95% CI = 0.770–1.730, *Z* = 0.700, *P *= 0.490).

**Figure 5:**
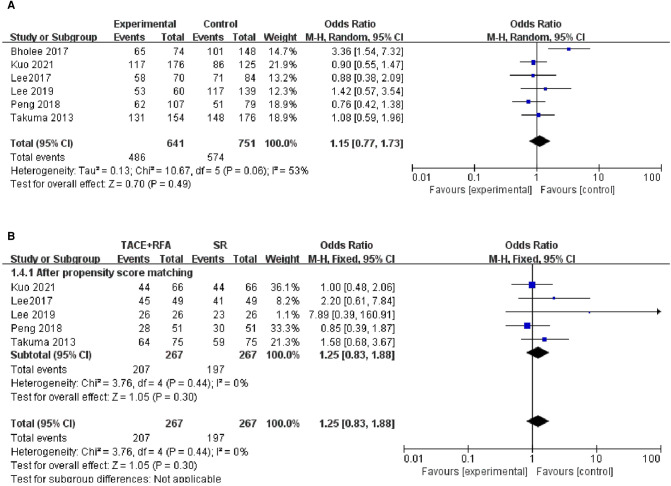
Comparison of (**A**) 1-year disease-free survival (DFS), (**B**) 1-year using DFS propensity score-matched.

Of these six studies, five studies were conducted using the propensity score-matched cohorts. There was no heterogeneity between the five studies (*I*^2^* *= 0%, *P *= 0.440), and a fixed-effects model was further used to combine the effect size. The results showed no significant difference in 1-year DFS between patients who received TACE + RFA and those who received SR (OR = 1.250, 95% CI = 0.830–1.880, *Z* = 1.050, *P *= 0.300). The above results are shown in Figure 5.

#### Three-Year DFS

Regarding 3-year DFS, six studies reporting 3-year DFS were included in this analysis. The heterogeneity test revealed heterogeneity among these studies (*I*^2^* *= 54%, *P *= 0.050). The results of the random-effects meta-analysis model showed that HCC patients who received SR had significantly better 3-year DFS compared with those who received TACE + RFA (OR = 0.700, 95% CI = 0.500–0.980, *Z* = 2.090, *P *= 0.040).

**Figure 6:**
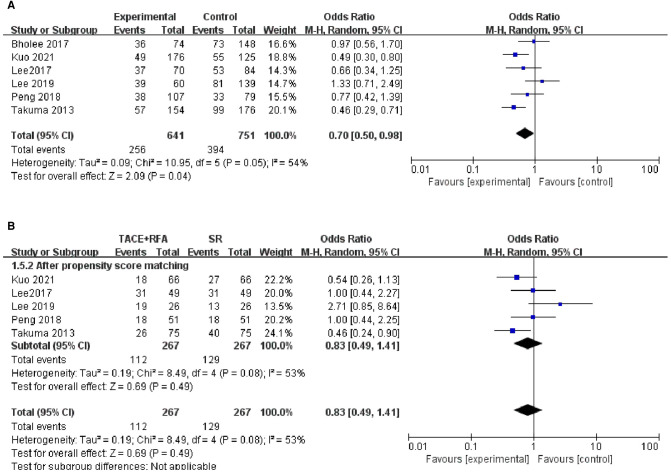
Comparison of (**A**) 3-year DFS, (**B**) 3-year using DFS propensity score-matched.

Of the six studies, five studies were conducted using the propensity score-matched cohorts. Since there was heterogeneity within these studies (*I*^2^* *= 53%, *P *= 0.080), a random-effects model was used to combine the effect size. The results of a subsequent meta-analysis showed that there was no significant difference in 3-year DFS between patients who received TACE + RFA and those who received SR (OR = 0.830, 95% CI = 0.490–1.410, *Z* = 0.690, *P *= 0.490). The above results are shown in Figure 6.

#### Five-Year DFS

Regarding 5-year DFS, only 6 of 12 studies reporting the 5-year DFS data were included in the following analysis. The heterogeneity test revealed the existence of heterogeneity among these studies (*I*^2^* *= 67%, *P *= 0.009). The results of the random-effects meta-analysis showed there was a statistically significant difference between the TACE + RFA group and the SR group (OR = 0.540, 95% CI = 0.360–0.830, *Z* = 2.860, *P *= 0.004), indicating that HCC patients who received SR had better 5-year DFS than those who received TACE + RFA.

**Figure 7:**
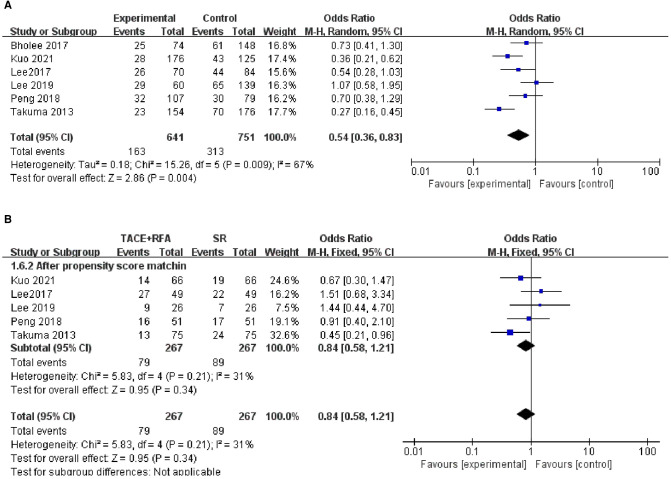
Comparison of (**A**) 5-year DFS, (**B**) 5-year using DFS propensity score-matched.

Of the six studies, five studies were conducted using the propensity score-matched cohorts. There was no heterogeneity in the five studies using the propensity score-matched cohorts (*I*^2^* *= 31%, *P *= 0.210). The results of the fixed-effects meta-analysis showed that there was no significant difference between the TACE + RFA group and SR group regarding the 5-year DFS (OR=0.840, 95% CI = 0.580–1.210, Z = 0.950, *P *= 0.340). The above results are shown in Figure 7.

#### Major Complication Rate

**Figure 8:**
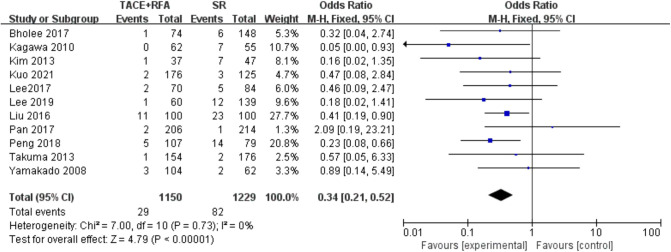
Comparison of major complication rate.

A total of 11 studies reporting the major complications were further included in the following analysis. The results of the heterogeneity test indicated that there was no heterogeneity among these studies (*I*^2^* *= 0%, *P *= 0.730). Therefore, the fixed-effects model was used for the following meta-analysis. The results showed that there was a statistically significant difference (OR = 0.340, 95% CI = 0.210–0.520, Z = 4.790, *P *< 0.00001), suggesting that HCC patients who received SR had a higher risk of major complications compared with those who received TACE + RFA (Figure 8).

#### Five-Year OS in Different Tumor Diameters

**Figure 9:**
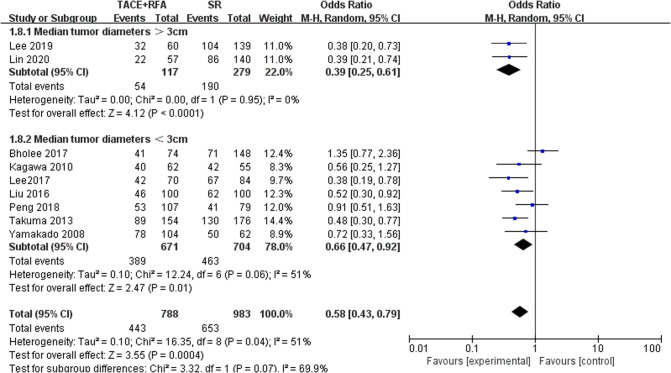
Comparison of 5-year OS in different tumor diameters.

According to the tumor diameter, nine studies were divided into two groups, and analysis was carried out separately (Figure 9). Based on the above subgroup analysis, the heterogeneity between the two groups was strong, which means that the tumor diameter of HCC patients will largely affect the analysis results. According to the analysis results, when the tumor diameter was longer than 3 cm, the 5-year OS rate was better when SR was selected.

#### Hospital Stay Time

**Figure 10:**
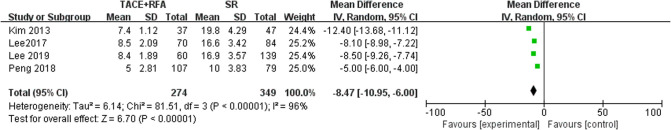
Hospital stay time.

Four studies reported hospital stay time. Since there was heterogeneity among the literature selected (*I*^2^* *= 96%, *P *< 0.00001), a random-effects model was used for meta-analysis. The results showed that there was a statistically significant difference (MD = −8.470, 95% CI = −10.950, −6.000, *Z* = 6.700, *P *< 0.00001), suggesting that HCC patients who received SR had a shorter hospital stay time compared with those who received TACE + RFA (Figure 10).

### Analysis of Publication Bias

**Figure 11:**
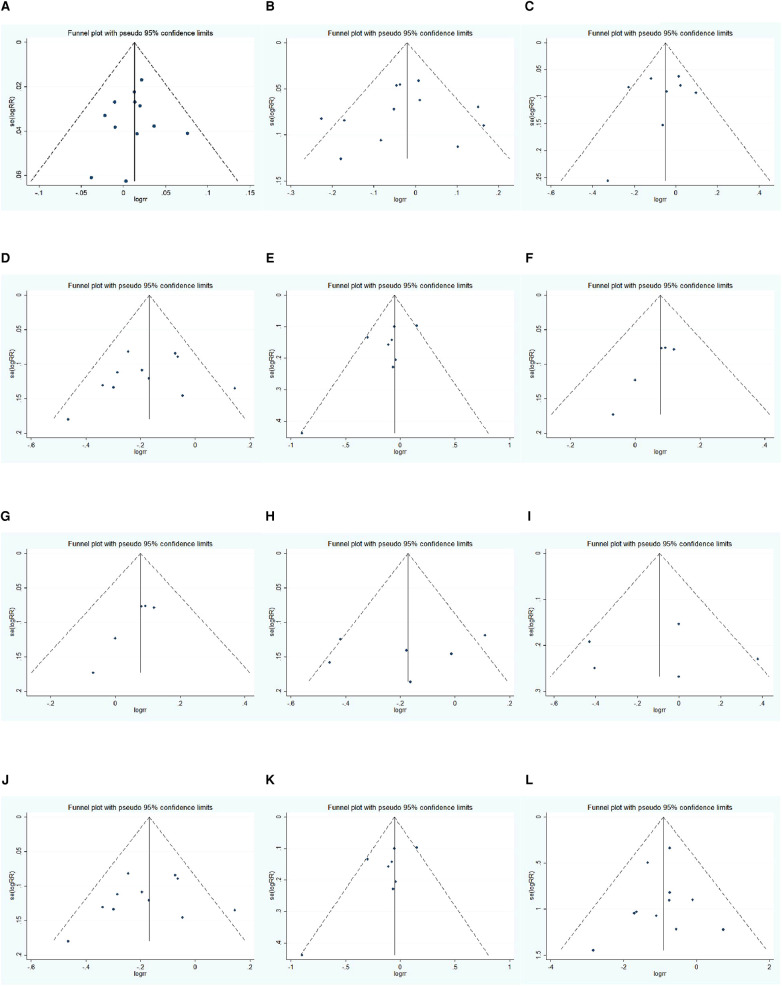
Funnel plot: (**A**) 1-year OS, (**B**) 3-year OS, (**C**) 3-year OS using propensity score-matched, (**D**) 5-year OS, (**E**) 5-year OS using propensity score-matched, (**F**) 1-year DFS, (**G**) 1-year DFS using propensity score-matched, (**H**) 3-year DFS, (**I**) 3-year DFS using propensity score-matched, (**J**) 5-year DFS, (**K**) 5-year DFS using propensity score-matched, (**L**) major complication rate.

The Egger test was conducted to evaluate the publication bias of the included literature. The results showed that the vast majority of studies have no publication bias in this meta-analysis: 1-year OS (*t *= 1.470, *P *= 0.173), 3-year OS before propensity score matching (*t *= 0.400, *P *= 0.698), 3-year OS after propensity score matching (*t *= 0.750, *P *= 0.483), 5-year OS before propensity score matching (*t *=* *−0.820, *P *= 0.435), 5-year OS after propensity score matching (*t *= −0.210, *P *= 0.840), 1-year DFS before propensity score matching (*t *= 1.150, *P *= 0.313), 1-year DFS after propensity score matching (*t *= 2.830, *P *= 0.066), 3-year DFS before propensity score matching (*t *= 2.540, *P *= 0.064), 3-year DFS after propensity score matching (*t *= 7.250, *P *= 0.005), 5-year DFS before propensity score matching (*t *= 2.010, *P *= 0.114), 5-year DFS after propensity score matching (*t *= 0.920, *P *= 0.427), and major complication (*t *= −0.090, *P *= 0.929). The funnel plot is shown in Figure 11.

## Discussion

The purpose of this meta-analysis was to assess and compare OS, DFS, and major complications of HCC patients who underwent TACE + RFA or SR alone, which offers objective considerations for physicians to select appropriate clinical treatment. Our meta-analysis showed the 1-year OS, 3-year OS, and 1-year DFS of HCC patients treated with TACE + RFA were not statistically significantly different from those treated with SR alone. Moreover, SR alone had superior long-term survival outcomes than TACE + RFA in terms of 5-year OS, 3-year DFS, and 5-year DFS, but TACE + RFA had a lower major complication rate than SR. However, there was significant heterogeneity between these studies regarding the 3-year OS, 5-year OS, 1-year DFS, 3-year DFS, and 5-year DFS. Therefore, we focused on studies using the propensity score-matched cohorts and performed another meta-analysis. The results revealed that SR had better 3- and 5-year OS than TACE + RFA in propensity score-matched cohort studies. However, there was no significant difference in 1-year DFS, 3-year DFS, and 5-year DFS among HCC patients who received TACE + RFA and SR treatment. For HCC patients with tumor diameter larger than 3 cm, SR had a higher long-term survival rate.

Compared with the meta-analysis conducted by other researchers, the results of Wang et al. and Gui et al. all showed that TACE + RFA had a higher incidence of major complication rate (20, 21). However, in the following research results, the results of several of our researchers were inconsistent: Wang et al.’s research results suggest that 1-year OS is higher in SR; the results of Gui et al. suggested that there was no significant difference in 5-year OS between TACE + RFA and SR. After our analysis, the reasons may be as follows: first, we included the latest studies and compared with the other two meta-analyses and we included more and newer studies; second, Wang et al.’s study did not use data after propensity score matching, which may result in a highly likely bias in meta-analysis results. Our study also carried out subgroup studies according to the HCC tumor diameter, which makes this study more convincing and clinically instructive.

RFA is an effective treatment option for HCC patients with a diameter smaller than 3 cm. Recent studies further showed that RFA in combination with TACE could effectively increase the ablation area of the tumor, thereby improving the feasibility of treating more large HCC tumors (22). The advantages of TACE + RFA for HCC treatment are as follows: (I) TACE prior to RFA can reduce the cooling effect of liver blood flow on RFA-mediated thermal coagulation; (II) TACE can cause tumor ischemia and edema by embolizing hepatic arterial flow, thereby increasing the area of tumor necrosis by subsequent RFA; (III) embolization of the peripheral portal vein around the tumor tissue by TACE can not only reduce the portal vein flow but also reduce the probability of the HCC invasion into the portal vein (23); and (IV) prior TACE treatment also reduces tumor size, an inherent limitation of RFA (24). A recent study by Liu et al. demonstrated that TACE combined with RFA significantly improves local tumor control and OS compared with TACE alone or RFA alone (8). In addition to the promising safety and remarkable synergistic effect on the treatment of HCC, TACE + RFA can further shorten the length of hospital stay, with high patient compliance (25).

SR is generally considered the most curative treatment for HCC (26), especially for patients with noncirrhotic HCC. Although extensive surgical resection can reduce the incidence of life-threatening complications in HCC patients (27), the recurrence rates after SR remain high and tends to increase in the total length of hospital stay, greatly increasing medical costs (28). Moreover, SR still has a statistically better survival advantage than RFA, especially for HCC nodules larger than 3 cm (29).

Multivariate analyses of risk factors in the study by Chai et al. showed that treatment modality and initial treatment response in HCC patients are significant predictors for OS and RFS, while recurrence after surgery is an independent prognostic factor for OS (30). In the retrospective study of 132 HCC patients who received TACE + RFA, tumor size, combined portal venous collateral circulation, alpha-fetoprotein, total bilirubin, and Child–Pugh grade are found to be independent risk factors that affect OS and the overall coexistence rate (31). According to the current literature, the main complication after TACE + RFA is embolism syndrome, while other serious complications include perforation of the gallbladder, skin burns, ectopic embolism, diaphragmatic fistula, intestinal necrosis, liver failure, intraperitoneal bleeding, and renal failure wait (10, 32).

Many patients with advanced cancer believe that quality of life (QoL) is as important as the length of life, which reinforces the importance of QoL evaluation in treating HCC patients (33). TACE is the most common palliative treatment for HCC, and common factors affecting the QoL of HCC patients treated with TACE include literacy, psychological support, access to medical care, liver dysfunction, treatment response, and multiple interventions (34). Older age at hepatectomy (≥65 years), recurrence during treatment, and type 2 changes in serum cholinesterase levels were associated with impaired QoL, and multivariate analysis showed that type 2 alteration of serum cholinesterase level was an independent risk factor for impaired QoL (35). Previous studies have shown that the postoperative QoL score of RFA is higher than that of SR, which may be due to minor liver injury in RFA (36). Since the length of hospital stay time is closely related to the QoL of HCC patients, the significantly shorter time in patients treated with TACE + RFA may contribute to a better QoL than those treated with SR.

Compared with previous meta-analyses, our study included more recent studies, larger sample sizes, more outcome indicators, and a certain amount of subgroup analysis. At the same time, this meta-analysis, for the first time, compared the results before and after the propensity score and compared the long-term survival rate of HCC patients with different tumor diameters, which is helpful in providing guidance for clinical researchers.

Also, our study has some limitations. First, most of the studies included were case–control studies, which may affect the reliability of the research data to a certain extent. Clinically, there is a difference between HCC patients targeted for TACE + RFA and HCC patients targeted for SR, which may account for the lack of RCTs. Second, since the results of these included studies were conducted in different years, there may also be biases due to the improvement of operators' skills and proficiency and the development of related medical devices. Third, although relevant recent studies have been included as many as possible, the number of studies in the final meta-analysis is still too small to conduct high-quality subgroup studies, such as the treatment efficacy of TACE + RFA and SR in different tumor stages. In clinical practice, researchers' recommendations based on patients' Child–Pugh scores and their own preferences may also lead to the eventual adoption of different treatment approaches (selection bias), which may also be one of the limitations of this study.

In conclusion, SR is superior to TACE + RFA in terms of long-term prognosis in HCC patients but may result in a higher risk of major complications. In addition, there was no significant difference in short-term survival outcomes between HCC patients who received TACE + RFA and those who received SR. More high-quality clinical studies are needed to strengthen the findings of this study.

## Data Availability

The original contributions presented in the study are included in the article/Supplementary Material, further inquiries can be directed to the corresponding author.
